# Comparative clinical performance of Alinity m HR HPV, cobas 4800 HPV, and cobas 6800 HPV for cervical cancer screening

**DOI:** 10.1128/spectrum.00589-25

**Published:** 2025-08-06

**Authors:** Shihai Huang, Luciana Girotto Gentil, Colleen Schmidt, Richard Cullum, Yan Zhang, Kevin Nelson, Danijela Lucic, Klara Abravaya

**Affiliations:** 1 Molecular Diagnostics of Abbott, Des Plaines, Illinois, USA; 2 Abbott Laboratories de Chile Ltda. 69746 , Santiago, Chile; Quest Diagnostics, Secaucus, New Jersey, USA

**Keywords:** cervical cancer, HPV test, primary screening, HPV genotyping

## Abstract

Primary high-risk human papillomavirus (HPV) testing is recommended for cervical cancer screening due to its sensitivity and high negative predictive value. Most of the cervical cancers are caused by HPV16 and HPV18, and their presence has been used to guide patient management. Here, we compared the clinical performance of the Alinity m HR HPV, cobas 4800 HPV, and cobas 6800 HPV assays in the context of cervical cancer screening. Clinical sensitivity and specificity were evaluated with 125 ≥CIN3 (cervical intraepithelial neoplasia grade 3) cases and 244 controls (≤CIN1). The genotype agreements between the assays were also evaluated for the case and control groups. The clinical sensitivities were 96.0% for Alinity m and cobas 6800 assays, and 95.2% for cobas 4800 assay. The clinical specificities observed were 67.6%, 68.0%, and 68.4% for Alinity m, cobas 6800, and cobas 4800 assays, respectively. Overall, the three HPV assays demonstrated similar clinical performance. In the ≥CIN3 group, genotype-specific positive agreement was ≥98.4% between Alinity m and cobas 4800 assays, and ≥85.7% between cobas 6800 and cobas 4800 assays. In the ≤CIN1 group, overall positive agreement among the three assays was ≥94.8%. This study showed similar clinical sensitivity and specificity for Alinity m HR HPV, cobas 4800 HPV, and cobas 6800 HPV assays. Alinity m was more specific in detecting HPV16 and HPV18, which could reduce unnecessary immediate referrals of women to colposcopy.

**IMPORTANCE:**

This study provides evidence that the Alinity m HR human papillomavirus (HPV) assay has similar clinical performance in comparison with the cobas 4800 HPV and cobas 6800 HPV, two widely used tests. Validation of HPV assays in a clinical setting is crucial to ensure that they can provide a balanced sensitivity and specificity for detecting high-grade cervical intraepithelial neoplasia, potentially improving patient management by enabling proper follow-up or treatment and avoiding unnecessary procedures.

## INTRODUCTION

Primary high-risk human papillomavirus (HPV) testing is the recommended strategy for cervical cancer screening because it is more sensitive in detecting high-grade cervical intraepithelial neoplasia and cancer (≥CIN3) at an early stage compared to traditional Pap tests (1). Implementation of primary HPV testing allows longer intervals between screenings due to its high negative predictive value for developing ≥CIN3 (2). Current guidelines recommend primary HPV testing every 5 years for individuals aged 25–65 (3–5).

Most HPV testing identifies a group of 14 carcinogenic HPV genotypes: 16, 18, 31, 33, 35, 39, 45, 51, 52, 56, 58, 59, 68, and 66. HPV66 is currently classified as possibly carcinogenic to humans (6). HPV16 and HPV18 are the most carcinogenic genotypes, responsible for approximately 70% of cervical cancers worldwide (7). HPV16 is highly associated with severe lesions and squamous cell carcinoma, the most common type of cervical cancer. HPV18 is more commonly present in adenocarcinomas (8). Accurate identification of these HPV genotypes is crucial for estimating the risk of progression to ≥CIN3 and for guiding clinical management, including the need for further diagnostic testing (9). As a result, high-risk HPV (hrHPV) tests with concurrent partial genotyping such as cobas HPV, or extended genotyping such as Alinity m HR HPV and BD Onclarity HPV, are progressively being incorporated into cervical cancer screening programs (10).

For this purpose, HPV tests must be clinically validated to meet the criteria required for use in cervical cancer screening, which include a balance between clinical sensitivity and specificity for disease detection (≥CIN3). The ability to differentiate HPV infections that are associated with high-grade lesions from those that are associated with transient infections can minimize unnecessary clinical follow-up procedures (11, 12).

The objective of this study was to evaluate and directly compare the clinical performance of the Alinity m HR HPV, cobas 4800 HPV, and cobas 6800 HPV tests in the context of cervical cancer screening. These FDA-approved assays are already clinically validated following international guidelines (13–15).

## MATERIALS AND METHODS

### Study population

A total of 369 cervical specimens from women ≥25 years of age stored in ThinPrep PreservCyt Solution (ThinPrep), participating in a multi-center, prospective study conducted in the United States, were selected for this evaluation. Of the 369 specimens, 125 were from women with histologically confirmed high-grade cervical disease (≥CIN3, also referred to as case group), including cases of squamous cell carcinoma, adenocarcinoma, and adenocarcinomas *in situ*; and 244 specimens were from (i) women not referred to colposcopy due to negative results on cytology, cobas 4800, and Alinity m assays, and (ii) women referred to colposcopy due to a positive HPV test result (from either Alinity m or cobas 4800) and/or cytology result ≥ASC-US, but without histologically confirmed high-grade cervical disease (≤CIN1, also referred to as the control group).

### HPV testing

#### Alinity m HR HPV assay

The Alinity m HR HPV assay (Abbott Molecular Inc., Des Plaines, IL, USA) for use on the Alinity m System is a qualitative assay that utilizes real-time polymerase chain reaction (PCR) to amplify and detect DNA sequences from 14 HPV genotypes. Genotypes HPV16, HPV18, and HPV45 are individually reported, and the remaining 11 genotypes are reported into group A (31, 33, 52, and 58) and group B (35, 39, 51, 56, 59, 66, and 68) (16).

#### cobas 4800 HPV test

The cobas 4800 HPV test (Roche Molecular Systems, Inc., Branchburg, NJ, USA) is a qualitative *in vitro* nucleic acid amplification test for individual detection of HPV16 and HPV18 genotypes and a combined detection of the additional 12 other HPV types (31, 33, 35, 39, 45, 51, 52, 56, 58, 59, 66, and 68). This assay can be used with the cobas 4800 System (17).

#### cobas 6800 HPV test

cobas HPV for use on the cobas 6800/8800 Systems (cobas HPV) (Roche Molecular Systems, Inc.) is a qualitative *in vitro* nucleic acid amplification test that detects 14 HPV genotypes, with individual identification of HPV16 and HPV18 genotypes. This assay can be used with cobas 6800/8800 Systems (18).

### Cytology and histology analysis

Cytology and histology results were obtained as previously described in detail (19).

Briefly, ThinPrep specimens were processed for cytology using the ThinPrep 2000 or ThinPrep 5000, and the results were graded using the 2014 Bethesda System (20). The results were grouped into normal (NILM), atypical squamous cells of undetermined significance (ASC-US), or any abnormal cytology other than ASC-US (>ASC-US).

For women referred to colposcopy, specimens were collected during the colposcopy visit following a standardized collection protocol: (i) an endocervical curettage was performed for all participants, (ii) biopsies were obtained on all visible lesions, and (iii) a single random biopsy of the visualized or partially visualized squamocolumnar junction was obtained if no lesion was visible.

### Statistical analysis

The clinical performance of the assays was evaluated by determining the clinical sensitivity for the case group (≥CIN3) and the clinical specificity for the control group (≤CIN1). The Alinity m and cobas 6800 genotyping results were compared to the results reported by the cobas 4800 test (reference test). The genotype results were defined hierarchically as follows: a positive result for HPV16 indicates the presence of HPV16 with or without the presence of HPV18 and 12 Other hrHPV, a positive result for HPV18 indicates the absence of HPV16 with or without the presence of 12 Other hrHPV, and a positive result for 12 Other HPV indicates the absence of HPV16 and HPV18 and the presence of any other hrHPV. The estimated percentages of agreement of positive and negative results, along with the 95% confidence intervals (CIs), were calculated.

Agreement among the HPV assays was evaluated using a composite comparator reference standard. For each assay, the composite result was determined by the concordant combined results of the two remaining comparator assays. Positive percent agreement (PPA) and negative percent agreement (NPA) with 95% CIs were calculated.

All data analyzes were performed using SAS software version 9.4 or higher (SAS, Cary, NC, USA).

## RESULTS

The study population consisted of 369 women, with mean and median ages of 40.4 and 38 (range: 25–73 years), respectively. For the control group (≤CIN1), the mean age was 42.1 and the median age was 40 (range: 25–72 years). For the case group (≥CIN3), the mean age was 37.1 years, and the median age was 34 years (range: 25–73 years).

The clinical performance of Alinity m HR HPV, cobas 6800 HPV, and cobas 4800 HPV assays is summarized in Table 1. Of the 125 ≥CIN3 cases, 120 were positive for Alinity m and cobas 6800, resulting in a clinical sensitivity of 96.0% for both assays. The cobas 4800 detected 119 ≥CIN3 cases, resulting in a sensitivity of 95.2%. Compared to cobas 4800, the relative clinical sensitivity for ≥CIN3 was 1.01 for Alinity m and for cobas 6800.

**TABLE 1:** Sensitivity for disease detection (≥CIN3) and specificity for ≤CIN1 of Alinity m, cobas 6800, and cobas 4800 in the total study population

HPV assay	Sensitivity for ≥CIN3		Specificity for ≤CIN1	
	% (95% CI)	*n*/*N*	% (95% CI)	*n*/*N*
Alinity m HR HPV	96.0 (91.0–98.3)	120/125	67.6 (61.5–73.2)	165/244
cobas 6800 HPV	96.0 (91.0–98.3)	120/125	68.0 (61.9–73.6)	166/244
cobas 4800 HPV	95.2 (89.9–97.8)	119/125	68.4 (62.4–73.9)	167/244

For clinical specificity, 244 cervical specimens, with ≤CIN1 histology or no lesion, were evaluated. The clinical specificity observed was 67.6%, 68.0%, and 68.4% for Alinity m, cobas 6800, and cobas 4800 assays, respectively. The relative clinical specificity for ≤CIN1 was 0.99 for Alinity m and cobas 6800, compared to cobas 4800. Overall, these three HPV assays demonstrated similar clinical sensitivity and specificity.

Overall positive agreement among the three assays was ≥98.3%, in the ≥CIN3 group (Tables 2 to 4). Genotype-specific evaluation demonstrated the highest genotype-specific positive agreement between cobas 4800 (reference assay) and Alinity m assays (≥98.4%), while the lowest genotype-specific positive agreement was observed between cobas 4800 and cobas 6800 assays (≥85.7%).

**TABLE 2:** Comparison of Alinity m and cobas 4800 results by genotypes for control and case groups

Alinity m HR HPV result	cobas 4800 HPV results					Total tested
	Overall HR HPV POS	HPV16	HPV18	12 Other HPV	Negative	
Cases (≥CIN3)						
Overall HR HPV POS	118	51	7	60	2	120
HPV16	51	51	0	0	0	51
HPV18	7	0	7	0	1	8
12 Other HPV^*a*^	60	0	0	60	1	61
Negative	1	0	0	1	4	5
Total	119	51	7	61	6	125
Percent agreement (95% CI) (*n*/*N*)	99.2 (95.4–99.9) (118/119)	100.0 (93.0–100.0) (51/51)	100.0 (64.6–100.0) (7/7)	98.4 (91.3–99.7) (60/61)	66.7 (30.0–90.3) (4/6)	–^*b*^
Controls (≤CIN1)						
Overall HR HPV POS	73	6	10	57	6	79
HPV16	5	5	0	0	0	5
HPV18	6	0	6	0	1	7
12 Other HPV^*a*^	62	1	4	57	5	67
Negative	4	0	0	4	161	165
Total	77	6	10	61	167	244
Percent agreement (95% CI) (*n*/*N*)	94.8 (87.4–98.0) (73/77)	83.3 (43.6–97.0) (5/6)	60.0 (31.3–83.2) (6/10)	93.4 (84.3–97.4) (57/61)	96.4 (92.4–98.3) (161/167)	–

^*a*^ 12 Other HPV consists of HPV45, Group A (HPV 31/33/52/58), and Group B (HPV 35/39/51/56/59/66/68) for Alinity m results.

^*b*^ ”–,” not applicable.

**TABLE 3:** Comparison of cobas 6800 and cobas 4800 results by genotypes for control and case groups

cobas 6800 HPV result	cobas 4800 HPV results					Total tested
	Overall HR HPV POS	HPV16	HPV18	12 Other HPV	Negative	
Cases (≥CIN3)						
Overall HR HPV POS	117	51	7	59	3	120
HPV16	52	51	1	0	0	52
HPV18	6	0	6	0	2	8
12 Other HPV^*a*^	59	0	0	59	1	60
Negative	2	0	0	2	3	5
Total	119	51	7	61	6	125
Percent agreement (95% CI) (*n*/*N*)	98.3 (94.1–99.5) (117/119)	100.0 (93.0–100.0) (51/51)	85.7 (48.7–97.4) (6/7)	96.7 (88.8–99.1) (59/61)	50.0 (18.8–81.2) (3/6)	–^*b*^
Controls (≤CIN1)						
Overall HR HPV POS	74	6	10	58	4	78
HPV16	7	6	0	1	1	8
HPV18	9	0	9	0	1	10
12 Other HPV^*a*^	58	0	1	57	2	60
Negative	3	0	0	3	163	166
Total	77	6	10	61	167	244
Percent agreement (95% CI) (*n*/*N*)	96.1 (89.2–98.7) (74/77)	100.0 (61.0–100.0) (6/6)	90.0 (59.6–98.2) (9/10)	93.4 (84.3–97.4) (57/61)	97.6 (94.0–99.1) (163/167)	–

^*a*^ 12 Other HPV consists of HPV45, Group A (HPV 31/33/52/58), and Group B (HPV 35/39/51/56/59/66/68) for Alinity m results.

^*b*^ ”–,” not applicable.

**TABLE 4:** Comparison of Alinity m and cobas 6800 results by genotypes for control and case groups

Alinity m HR HPV result	cobas 6800 HPV results					Total tested
	Overall HR HPV POS	HPV16	HPV18	12 Other HPV	Negative	
Cases (≥CIN3)						
Overall HR HPV POS	118	52	7	59	2	120
HPV16	51	51	0	0	0	51
HPV18	8	1	7	0	0	8
12 Other HPV^*a*^	59	0	0	59	2	61
Negative	2	0	1	1	3	5
Total	120	52	8	60	5	125
Percent agreement (95% CI) (*n*/*N*)	98.3 (94.1–99.5) (118/120)	98.1 (89.9–99.7) (51/52)	87.5 (52.9–97.8) (7/8)	98.3 (91.1–99.7) (59/60)	60.0 (23.1–88.2) (3/5)	–^*b*^
Controls (≤CIN1)						
Overall HR HPV POS	74	7	10	57	5	79
HPV16	5	5	0	0	0	5
HPV18	7	0	7	0	0	7
12 Other HPV^*a*^	62	2	3	57	5	67
Negative	4	1	0	3	161	165
Total	78	8	10	60	166	244
Percent agreement (95% CI) (*n*/*N*)	94.9 (87.5–98.0) (74/78)	62.5 (30.6–86.3) (5/8)	70.0 (39.7–89.2) (7/10)	95.0 (86.3–98.3) (57/60)	97.0 (93.1–98.7) (161/166)	–

^*a*^ 12 Other HPV consists of HPV45, Group A (HPV 31/33/52/58), and Group B (HPV 35/39/51/56/59/66/68) for Alinity m results.

^*b*^ "–,” not applicable.

In the ≤CIN1 group, overall positive agreement among the three assays was ≥94.8% (Tables 2 to 4). Genotype-specific evaluation demonstrated that HPV16 had 100% (6/6) agreement between cobas 4800 and cobas 6800, while the lowest agreement 62.5% (5/8) was between Alinity m and cobas 6800 (Tables 3 and 4). HPV18 agreement rates were 60% (6/10) between Alinity m and cobas 4800, 70% (7/10) between Alinity m and cobas 6800, and 90% (9/10) between cobas 6800 and cobas 4800. The overall agreement for the 12 Other results was ≥93.4% (Tables 2 to 4).

Overall, the clinical false-positive rates, HPV detection in the control group, were similar among the assays, 32.4% (79/244), 32.0% (78/244), and 31.6% (77/244) for Alinity m, cobas 6800, and cobas 4800, respectively. Compared to Alinity m, cobas 6800, and cobas 4800 assays exhibited more clinical false-positive results for HPV16 and HPV18. The cobas 6800 and cobas 4800 assays showed 7.4% (18/244) and 6.6% (16/244) clinical false-positive results, respectively, while Alinity showed 4.9% (12/244). For non-16/18 HPV, Alinity m showed slightly more clinical false-positive results than the cobas HPV assays. Alinity m, cobas 4800, and cobas 6800 showed 27.5% (67/244), 25.0% (61/244), and 24.6% (60/244) clinical false-positive results, respectively.

In this study, we could not compare the performance of HPV45, but Alinity m HR HPV detected HPV45 in a total of 11 samples, including 7 from ≥CIN3 subjects and 4 from ≤CIN1 subjects. The majority of these samples had a single HPV45 infection by Alinity m. They were reported as 12 Other HPV by cobas 6800, while cobas 4800 reported 10 out of 11 as single infection and one as mixed infection.

Compositive comparator performance was evaluated for the control and case groups. Data were included in the analysis only when two concordant results were obtained from the comparator assays. The overall PPA for the case group and NPA for the control group showed similar performance among the assays as presented in Table S1.

Discordant results between any two assays were observed in 33 specimens, as shown in Table 5. Of the 22 discordant results in the ≤CIN1 group, five were Alinity m positive for non-16/18 HPV and cobas 6800 negative. Four results were Alinity m negative and cobas 6800 positive (three Other HR HPV and one HPV16). Among the partially discordant results, six were reported as non-16/18 HPV on Alinity m (Other A or Other B groups), while on cobas 4800 and/or 6800, they were reported as HPV16 or HPV18, in addition to the non-16/18 HPV. The discordant results for detecting ≥CIN3 were found to be evenly distributed among the three assays. The HPV16 (specimen 5) and HPV18 (specimen 7) results that were missed by cobas 4800 and Alinity m showed a late cycle number beyond the limit of detection (data not shown).

**TABLE 5:** Discordant results of Alinity m, cobas 4800, and cobas 6800 in the total study population

Specimen no. (decodified)	Alinity m result^*a*^	cobas 6800 result^*b*^	cobas 4800 result^*b*^	Frequency
Cases (≥CIN3)				
1	HPV16	HPV16	HPV16; Other	1
2–4	HPV16	HPV16; Other	HPV16; Other	3
5	HPV18	HPV16; HPV18	HPV18	1
6	HPV18	HPV18	Negative	1
7	Negative	HPV18	Negative	1
8	Negative	Other	Other	1
9	Other A	Negative	Other	1
10	Other A	Other	Negative	1
11	Other B	Negative	Other	1
Controls (≤CIN1)				
12	HPV16	HPV16; HPV18	HPV16	1
13–14	HPV16	HPV16; Other	HPV16; Other	2
15	HPV18	HPV18	Negative	1
16	HPV45; Other A; Other B	Other	HPV18; Other	1
17	Negative	HPV16	Negative	1
18–20	Negative	Negative	Other	3
21–22	Negative	Other	Negative	2
23	Negative	Other	Other	1
24	Other A	HPV16; Other	Other	1
25	Other A	Negative	Negative	1
26	Other A; Other B	HPV16; Other	HPV16; Other	1
27–29	Other B	HPV18; Other	HPV18; Other	3
30–33	Other B	Negative	Negative	4

^*a*^ Other A: HPV 31/33/52/58; Other B: HPV 35/39/51/56/59/66/68.

^*b*^ Other: HPV 31/33/35/39/45/51/52/56/58/59/66/68.

## DISCUSSION

Alinity m HR HPV assay, cobas 4800 HPV, and cobas 6800/8800 HPV tests have been clinically validated and are used in primary HPV testing for cervical cancer screening (13–15, 21–24). Recently, the cobas 4800 HPV, RealTime High-Risk HPV (Abbott), BD Onclarity HPV (BD Diagnostics), and Anyplex II HPV HR Detection (Seegene) tests were accepted by a 2024 contemporary international consensus as a second-generation comparator test for the clinical validation of HPV tests using clinician-collected cervical specimens for cervical cancer screening (12).

In this study, we demonstrated that Alinity m, cobas 4800, and cobas 6800 tests have comparable clinical performance. The relative sensitivity for ≥CIN3 was 1.01 for Alinity m or cobas 6800 versus cobas 4800 test, while the relative specificity for ≤CIN1 was 0.99, meeting the international validation criteria for effective use as primary HPV tests, including the new criteria established for the second generation comparators test (0.95 relative clinical sensitivity) (11, 12). Similar relative performance was observed in previous studies for Alinity m versus cobas 4800 in a population-based screening setting (22, 23), and for cobas 4800 versus cobas 6800 (15, 21, 24). Additionally, the assays were evaluated using compositive comparator analysis, which also demonstrated comparable performance among them.

Despite the similar relative specificity observed among the evaluated tests, the low absolute specificity may be attributed to the broader inclusion criteria applied to the control group. In the present study, the control group included not only women with negative cytology results, but also those with cytological findings ≥ASC-US, who were referred for colposcopy evaluation and presented a single histological diagnosis of ≤CIN1. This differs from other studies that included only women with two consecutive negative cytology results or without histological evidence of >CIN2 within 6 months of follow-up (15, 22).

Although the overall performance differences among the assays were not statistically significant, some discrepant results were observed, including the HPV genotype agreements. The cobas 6800 and cobas 4800 tests exhibited more clinical false-positive results for HPV16 and HPV18 than the Alinity m assay, which can impact the clinical management.

In addition, cobas 6800 exhibited more clinical false-positive results for HPV16 and HPV18 than cobas 4800. This higher level of detection of HPV16 and HPV18 was consistent with results by Frayle et al. which showed 31 clinical false-positive HPV16 or HPV18 results in 925 <CIN2 cases (3.4%) by cobas 6800 in comparison with 18 clinical false-positive results by cobas 4800 (1.9%) (21).

Detection of clinical false-positive results can lead to unnecessary and invasive follow-up procedures, such as colposcopy, biopsy, and treatment, which carry their own risks. It also impacts patient quality of life by causing anxiety from receiving an hrHPV positive result and discomfort from subsequent clinical procedures with additional costs (5).

On the other hand, compared to cobas 6800, Alinity m showed more clinical false-positive results for non-HPV16/18 HPV. Although it is still crucial to monitor these genotypes, they allow a less invasive management strategy. For non-16/18 HPV results, it has been proposed a reflex cytology and referral to colposcopy only if cytology result is ≥ASC-US. If cytology result is negative, HPV testing would be performed after 12 months (25).

In addition to the individual detection of HPV16 and HPV18, the Alinity m assay also individually detects HPV45, which is the third most common HPV type in cervical cancer and strongly associated with cervical adenocarcinoma (26). Together with HPV18, HPV45 is responsible for 44% of adenocarcinomas (8, 27). It has been indicated that women with HPV positive for these three genotypes should be considered for colposcopy (7). In this study, HPV16, HPV18, and HPV45 were detected in 40.8% (51/125), 6.4% (8/125), and 5.6% (7/125) of ≥CIN3 cases, respectively. Although the number of carcinoma cases was limited, at least one of these three genotypes was present in 91.7% (11/12) of those cases, supporting the clinical utility of detecting these genotypes. Previous cross-sectional studies have demonstrated that women who are HPV18 or HPV45 positive exhibit a higher prevalence of invasive squamous cell cancer, especially adenocarcinoma, compared to those infected with other hrHPV genotypes (non-16/18/45 HPV) (28–30). Therefore, HPV assays that report HPV45 individually may be used effectively in clinical management to detect occult glandular neoplasia associated with HPV45 (30).

Overall, this study showed a high concordance in the clinical performance of Alinity m HR HPV assay, cobas 4800 HPV, and cobas 6800 HPV assays. In this clinical population, Alinity m would reduce the number of women referred immediately to colposcopy and help avoid unnecessary procedures, due to lower rate of clinical false-positive results for HPV16 and HPV18 in cases of ≤CIN1.

## SUPPLEMENTAL MATERIAL

Table S1 spectrum.00589-25-s0001.pdf Performance of Alinity m, cobas 4800, and cobas 6800 assays using a composite comparator.

ASM does not own the copyrights to Supplemental Material that may be linked to, or accessed through, an article. The authors have granted ASM a non-exclusive, world-wide license to publish the Supplemental Material files. Please contact the corresponding author directly for reuse.

## References

[1] Rizzo AE, Feldman S. 2018. Update on primary HPV screening for cervical cancer prevention. Curr Probl Cancer 42:507–520. doi:10.1016/j.currproblcancer.2018.06.013 30146348

[2] Elfström KM, Smelov V, Johansson ALV, Eklund C, Nauclér P, Arnheim-Dahlström L, Dillner J. 2014. Long term duration of protective effect for HPV negative women: follow-up of primary HPV screening randomised controlled trial. BMJ 348:g130. doi:10.1136/bmj.g130 24435414 PMC3898575

[3] USPSTF. 2018 USPSTF recommendation: cervical cancer: screening, on United States preventive services taskforce. Available from: https://www.uspreventiveservicestaskforce.org/uspstf/recommendation/cervical-cancer-screening#fullrecommendationstart

[4] ACOG. 2024. Updated cervical cancer screening guidelines, on American college of obstetricians and gynecologists. Available from: https://www.acog.org/clinical/clinical-guidance/practice-advisory/articles/2021/04/updated-cervical-cancer-screening-guidelines

[5] Fontham ETH, Wolf AMD, Church TR, Etzioni R, Flowers CR, Herzig A, Guerra CE, Oeffinger KC, Shih Y-CT, Walter LC, Kim JJ, Andrews KS, DeSantis CE, Fedewa SA, Manassaram-Baptiste D, Saslow D, Wender RC, Smith RA. 2020. Cervical cancer screening for individuals at average risk: 2020 guideline update from the American Cancer Society. CA Cancer J Clin 70:321–346. doi:10.3322/caac.21628 32729638

[6] IARC. 2022. Cervical cancer screening: IARC handbooks of cancer prevention. Vol. 18. IARC Publications.

[7] Ye Y, Jones T, Wang T, Zeng X, Liu Y, Zhao C. 2024. Comprehensive overview of genotype distribution and prevalence of human papillomavirus in cervical lesions. Gynecol Obstet Clin Med 4:e000005. doi:10.1136/gocm-2024-000005 38650896 PMC11034807

[8] de Sanjose S, Quint WG, Alemany L, Geraets DT, Klaustermeier JE, Lloveras B, Tous S, Felix A, Bravo LE, Shin H-R, et al.. 2010. Human papillomavirus genotype attribution in invasive cervical cancer: a retrospective cross-sectional worldwide study. Lancet Oncol 11:1048–1056. doi:10.1016/S1470-2045(10)70230-8 20952254

[9] Demarco M, Hyun N, Carter-Pokras O, Raine-Bennett TR, Cheung L, Chen X, Hammer A, Campos N, Kinney W, Gage JC, Befano B, Perkins RB, He X, Dallal C, Chen J, Poitras N, Mayrand MH, Coutlee F, Burk RD, Lorey T, Castle PE, Wentzensen N, Schiffman M. 2020. A study of type-specific HPV natural history and implications for contemporary cervical cancer screening programs. EClinicalMedicine 22:100293. doi:10.1016/j.eclinm.2020.100293 32510043 PMC7264956

[10] Poljak M, Oštrbenk Valenčak A, Cuschieri K, Bohinc KB, Arbyn M. 2024. 2023 global inventory of commercial molecular tests for human papillomaviruses (HPV). J Clin Virol 172:105671. doi:10.1016/j.jcv.2024.105671 38518504

[11] Meijer CJLM, Berkhof J, Castle PE, Hesselink AT, Franco EL, Ronco G, Arbyn M, Bosch FX, Cuzick J, Dillner J, Heideman DAM, Snijders PJF. 2009. Guidelines for human papillomavirus DNA test requirements for primary cervical cancer screening in women 30 years and older. Int J Cancer 124:516–520. doi:10.1002/ijc.24010 18973271 PMC2789446

[12] Arbyn M, Cuschieri K, Bonde J, Schuurman R, Cocuzza C, Broeck DV, Zhao F, Rezhake R, Gultekin M, de Sanjosé S, Canfell K, Hawkes D, Saville M, Hillemanns P, Dillner J, Berkhof J, Prétet J, Gheit T, Clifford G, Basu P, Almonte M, Wentzensen N, Poljak M. 2024. Criteria for second generation comparator tests in validation of novel HPV DNA tests for use in cervical cancer screening. J Med Virol 96:e29881. doi:10.1002/jmv.29881 39221498

[13] Heideman DAM, Hesselink AT, Berkhof J, van Kemenade F, Melchers WJG, Daalmeijer NF, Verkuijten M, Meijer CJLM, Snijders PJF. 2011. Clinical validation of the cobas 4800 HPV test for cervical screening purposes. J Clin Microbiol 49:3983–3985. doi:10.1128/JCM.05552-11 21880968 PMC3209101

[14] Oštrbenk Valenčak A, Šterbenc A, Seme K, Poljak M. 2019. Alinity m HR HPV assay fulfills criteria for human papillomavirus test requirements in cervical cancer screening settings. J Clin Microbiol 58:e01120-19. doi:10.1128/JCM.01120-19 31666369 PMC6935899

[15] Saville M, Sultana F, Malloy MJ, Velentzis LS, Caruana M, Ip ELO, Keung MHT, Canfell K, Brotherton JML, Hawkes D. 2019. Clinical validation of the cobas HPV test on the cobas 6800 system for the purpose of cervical screening. J Clin Microbiol 57:e01239-18. doi:10.1128/JCM.01239-18 30463896 PMC6355513

[16] Molecular A. 2023. HR HPV AMP Kit package insert. Ref 09N15-095 (53-608175/R1). Des Plaines, IL, USA Abbott Molecular Inc

[17] Molecular R. 2015. cobas HPV test package insert. 05641268001-12EN Doc Rev. 12.0. Branchburg, NJ Roche Molecular Systems, Inc

[18] Molecular R. 2020. cobas HPV for use on the cobas 6800/8800 Systems (cobas HPV) package insert. 07998031001-01EN Doc Rev. 1.0. Branchburg, NJ Roche Molecular Systems, Inc

[19] Stoler M, Cullum R, Lucic D, Wright T Jr. 2025. Alinity m HR HPV assay: United States clinical trial design and high-risk human papillomavirus prevalence. J Low Genit Tract Dis 29:235–238. doi:10.1097/LGT.0000000000000900 40455582 PMC12188798

[20] Nayar R, Wilbur DC. 2015. The bethesda system for reporting cervical cytology: definitions, criteria, and explanatory notes. 3rd ed. Springer, Cham, Switzerland.

[21] Frayle H, Gori S, Rizzi M, Graziani BN, Vian E, Giorgi Rossi P, Del Mistro A. 2019. HPV testing for cervical cancer screening: technical improvement of laboratory logistics and good clinical performance of the cobas 6800 in comparison to the 4800 system. BMC Womens Health 19:47. doi:10.1186/s12905-019-0743-0 30909894 PMC6434866

[22] Dhillon SK, Oštrbenk Valenčak A, Xu L, Poljak M, Arbyn M. 2021. Clinical and analytical evaluation of the alinity m HR HPV assay within the VALGENT-3 framework. J Clin Microbiol 59:e00286-21. doi:10.1128/JCM.00286-21 33731413 PMC8316144

[23] Oštrbenk Valenčak A, Bertram A, Gröning A, Poljak M. 2021. Comparison of the clinical and analytical performance of Alinity m HR HPV and cobas 4800 HPV assays in a population-based screening setting. J Clin Virol 140:104851. doi:10.1016/j.jcv.2021.104851 34020361

[24] Sundström K, Lamin H, Dillner J. 2021. Validation of the cobas 6800 human papillomavirus test in primary cervical screening. PLoS One 16:e0247291. doi:10.1371/journal.pone.0247291 33606755 PMC7894810

[25] Teixeira JC, Vale DB, Campos CS, Polegatto I, Bragança JF, Discacciati MG, Zeferino LC. 2024. Transition from opportunistic cytological to organized screening program with DNA-HPV testing detected prevalent cervical cancers 10 years in advance. Sci Rep 14:20761. doi:10.1038/s41598-024-71735-2 39237756 PMC11377760

[26] ASCCP. 2024. Enduring guidelines extended genotyping evidence summary and proposed recommendations. Available from: https://www.asccp.org/Assets/80742b9b-2b64-4140-a81c-981eaab5ee26/638467215678330000/enduring-guidelines-extended-genotyping-summary-032224-pdf

[27] Wei F, Georges D, Man I, Baussano I, Clifford GM. 2024. Causal attribution of human papillomavirus genotypes to invasive cervical cancer worldwide: a systematic analysis of the global literature. Lancet 404:435–444. doi:10.1016/S0140-6736(24)01097-3 39097395

[28] Dahlström LA, Ylitalo N, Sundström K, Palmgren J, Ploner A, Eloranta S, Sanjeevi CB, Andersson S, Rohan T, Dillner J, Adami H-O, Sparén P. 2010. Prospective study of human papillomavirus and risk of cervical adenocarcinoma. Int J Cancer 127:1923–1930. doi:10.1002/ijc.25408 20473898 PMC2930102

[29] Sundström K, Eloranta S, Sparén P, Arnheim Dahlström L, Gunnell A, Lindgren A, Palmgren J, Ploner A, Sanjeevi CB, Melbye M, Dillner J, Adami H-O, Ylitalo N. 2010. Prospective study of human papillomavirus (HPV) types, HPV persistence, and risk of squamous cell carcinoma of the cervix. Cancer Epidemiol Biomarkers Prev 19:2469–2478. doi:10.1158/1055-9965.EPI-10-0424 20671136 PMC2952359

[30] Stoler MH, Wright TC, Parvu V, Yanson K, Eckert K, Kodsi S, Cooper C. 2019. HPV testing With 16, 18, and 45 genotyping stratifies cancer risk for women with normal cytology. Am J Clin Pathol 151:433–442. doi:10.1093/ajcp/aqy169 30649177 PMC6396747

