# Supplementary material for: Comparative clinical performance of Alinity m HR HPV, cobas 4800 HPV, and cobas 6800 HPV for cervical cancer screening
Source: Microbiol Spectr. 2025 Aug 6;13(9):e00589-25. doi: 10.1128/spectrum.00589-25 (PMC12403635; doi:10.1128/spectrum.00589-25)
Supplement: Table S1 — Performance of Alinity m, cobas 4800, and cobas 6800 assays using a composite comparator. [file spectrum.00589-25-s0001.pdf]

Supplemental Table 1. Performance of Alinity m, cobas 4800 and cobas 6800 assays using a composite comparator (CC).

	N	CC+ Assay+	CC+ Assay-	CC- Assay+	CC- Assay-	PPA (%)		NPA (%)	
						Estimate (95% CI)	n / N	Estimate (95% CI)	n / N
**Cases (≥ CIN3)**									
Alinity m vs. CC	120	116	1	0	3	99.1 (95.3-99.8)	99.1 (116/117)	100.0 (43.9-100.0)	100.0 (3/3)
cobas 4800 vs. CC	121	116	2	0	3	98.3 (94.0-99.5)	98.3 (116/118)	100.0 (43.9-100.0)	100.0 (3/3)
cobas 6800 vs. CC	122	116	2	1	3	98.3 (94.0-99.5)	98.3 (116/118)	75.0 (30.1-95.4)	75.0 (3/4)
**Controls (≤ CIN1)**									
Alinity m vs. CC	237	73	1	5	158	98.6 (92.7-99.8)	98.6 (73/74)	96.9 (93.0-98.7)	96.9 (158/163)
cobas 4800 vs. CC	235	73	1	3	158	98.6 (92.7-99.8)	98.6 (73/74)	98.1 (94.7-99.4)	98.1 (158/161)
cobas 6800 vs. CC	234	73	0	3	158	100.0 (95.0-100.0)	100.0 (73/73)	98.1 (94.7-99.4)	98.1 (158/161)
